# Characteristics of the Users of Troubled Desire, a Web-Based Self-management App for Individuals With Sexual Interest in Children: Descriptive Analysis of Self-assessment Data

**DOI:** 10.2196/22277

**Published:** 2021-02-19

**Authors:** Miriam Schuler, Hannes Gieseler, Katharina W Schweder, Maximilian von Heyden, Klaus M Beier

**Affiliations:** 1 Department of Health and Human Sciences Institute of Sexology and Sexual Medicine Charité-Universitätsmedizin Berlin Berlin Germany

**Keywords:** pedophilia, hebephilia, child sexual offenses, child sexual abuse material, web-based assessment, web-based treatment

## Abstract

**Background:**

Despite the high prevalence of child sexual offenses and the increasing amounts of available child sexual abuse material, there is a global shortage of preventive interventions focusing on individuals at risk of sexual offending. The web-based app Troubled Desire aims to address this shortage by offering self-assessments and self-management training modules in different languages to individuals with sexual interests in prepubescent and early pubescent children (ie, those with pedophilic and hebephiliac sexual interest, respectively).

**Objective:**

The aim of this study was to describe the characteristics of the users of the Troubled Desire app.

**Methods:**

The fully completed self-assessment data gathered within the first 30 months of this study from October 25, 2017 to April 25, 2020 were investigated. The main outcome measures were (1) sociodemographic information and (2) sexual interests and sexual behaviors of the users of Troubled Desire.

**Results:**

The self-assessment was completed by 4161 users. User accesses were mainly from Germany (2277/4161, 54.7%) and the United States (474/4161, 11.4%). Approximately 78.9% (3281/4161) of the users reported sexual interest in children; these users were significantly more likely to report distress and trouble owing to their sexual interest. Further, child sexual offenses and consumption of child sexual abuse material were significantly more common among users with sexual interest in children than among users with no sexual interest in children. Additionally, the majority of the offenses were not known to legal authorities.

**Conclusions:**

The Troubled Desire app is useful in reaching out to individuals with sexual interest in prepubescent and early pubescent children. However, future research is warranted to understand the prospective relevance of the Troubled Desire app in the prevention of child sexual offending.

## Introduction

### Background

Child sexual offenses and consumption of child sexual abuse material (CSAM) are global problems of great magnitude. A recent meta-analysis of 55 studies from 24 countries revealed prevalence estimates of child sexual offenses ranging from 8% to 31% for girls and from 3% to 17% for boys [1]. Additionally, the increasing use of the internet has resulted in considerable growth in the available CSAM content. In 2010, the Internet Watch Foundation identified 1351 webpages providing CSAM. These numbers escalated to 13,182 in 2013 and to 105,047 in 2018—more than a seven-fold increase within 8 years [2]. Experiencing sexual abuse during childhood can have profound negative short-term and long-term effects on the psychological and physiological well-being of children, including substance abuse, depression, suicidal tendencies, sexualized and risk-taking behavior, and increased risk for revictimization [3]. Self-evidently, the high number of sexually abused children and the harmful effects on their health status crucially demand prevention interventions to prevent child sexual victimization. A target group that is particularly suitable for prevention approaches are individuals with pedophilia (sexual interest in prepubescent children) [4] and individuals with hebephilia (sexual interest in early pubescent children) [5]. Pedophilic and hebephiliac sexual interests are not prerequisite for engaging in child sexual offenses or for the consumption of CSAM. However, both pedophilia and hebephilia can be regarded as the major risk factors for committing sexual offenses against children [6,7]. Pedophilic and hebephiliac individuals account for approximately 40%-50% of the officially registered child sexual offenses [8]. The consumption of CSAM seems to be a stronger indicator of pedophilic and hebephiliac sexual interest than committing sexual offenses against children [9].

In Germany, it has been possible to establish a network of outpatient treatment services for self-identified adolescents and adults with sexual interests in prepubescent and early pubescent children, regardless of their offending history [10,11]. The aim of these outpatient treatment services is to support these individuals in guaranteeing continuous sexual self-control to prevent initial or repeated child sexual offenses and consumption of CSAM. These treatment services have been made possible by the pledge of confidentiality for therapists under the German law. Unlike many other countries, Germany does not have a mandatory reporting policy; therefore, health care professionals (ie, therapists, physicians, or social workers) are not obliged to report actual or suspected past offenses. Hence, therapeutic offers are also provided to individuals who voluntarily seek help concerning their sexual attraction to children, even if they have offended in the past. Further, in cases of acute danger to children, the treatment services act according to a structured child protection procedure. To terminate the acute danger, the procedure ranges from implementation of specific and feasible strategies (eg, involvement of third parties to enhance social control, move out of the shared apartment) to consideration of medical interventions to reduce sexual impulses and ultimately, to admission to a psychiatry clinic [12]. In many other countries, such treatment services are not possible. There are mandatory reporting laws, which require certain professionals to report actual or suspected cases of child sexual offense and the use of CSAM to government authorities. Anyone who does not report actual or suspected cases will be liable to prosecution [13]. Individuals at risk of sexual offending or with history of offending behavior do not, therefore, receive therapeutic support without being reported to the authorities, which may discourage them from seeking help to prevent future sexual offending behavior [14]. However, it must be considered that in-person treatment is not suitable for all individuals in need of help. Besides practical barriers, including long waiting lists, distance to treatment services, travel expenses, or time conflicts between treatment availability and daily work, other emotional/psychological barriers such as shame or fear of stigmatization might prevent individuals from seeking help [15]. Furthermore, the abovementioned German outpatient treatment services are designed for individuals who are not under judicial supervision due to a sexual offense. Individuals who have recently been reported to governmental authorities or with criminal proceedings in process are not included in the program.

Summing up, we are facing a clear undersupply of preventive interventions focusing on relapse or prevention of child sexual offending. An option for individuals who want to seek help but are unable to receive in-person treatment (due to the reasons stated above) is the use of web-based interventions. Web-based interventions can be accessed anytime and anywhere, and anonymity can easily be ensured, which may encourage honesty and openness. There is a diverse range of web-based interventions. Some are solely psychoeducative, while others offer practical training modules. Some interventions also offer professional guidance and personalized feedback, while others are merely self-help websites [16,17]. Several meta-analyses have supported the application of web-based interventions for a variety of psychological disorders [18-25]. A meta-analysis performed on 92 studies investigating the effectiveness of web-based psychological interventions found a medium effect for such interventions (mean weighted effect size of 0.53), which is comparable to the effect sizes of in-person treatments [26].

In recent years, web-based interventions targeting sexology and forensic psychiatry have become established [27,28]; however, the offer is still scarce. The Candeo Treatment Program [29] offers a comprehensive web-based program to help individuals struggling with addictive pornography use. Preliminary results of the cognitive behavioral therapy approach of Candeo were promising as there was a reduction in pornography consumption and masturbation frequency after the treatment was introduced. Additionally, participants reported that Candeo was more helpful than the other treatments they had tried [30]. Kernsmith and Kernsmith [31] examined the process and effectiveness of a self-help group for recovering sex offenders. This group was moderated by 2 nonprofessionals, who themselves had offended in the past, and this group was created to provide a space to discuss feelings and struggles in a safe environment. Preliminary results indicated that this group had a reduction in the risk factors associated with recidivism (eg, cognitive distortions, compulsive behaviors, obsessive thoughts).

Web-based interventions specifically designed for individuals with sexual interest in children barely exist. To our knowledge, there is 1 German-speaking (Gemeinsam statt allein, Together instead of alone, in English [32]) and 2 English-speaking (VirPed [33] and B4uact [34]) peer-support forums specifically designed for individuals with sexual interest in children. These forums aim to provide peer support and information about the available resources on how to prevent sexual offenses and lead a content life. Furthermore, regarding professional web-based programs, apart from Troubled Desire, there are only 2 other self-help programs for individuals with sexual interest in children: (1) Help Wanted [35] and (2) the self-help program of Stop it now! [36]. Help Wanted was recently developed (published online in 2020) at the Moore Center for the Prevention of Child Sexual Abuse at Johns Hopkins Bloomberg School of Public Health, and it provides video modules and additional material to help individuals manage their sexual attraction and build a healthy nonoffending life. Stop it now! is a project of the UK-wide child protection charity Lucy Faithfull Foundation and offers live chats, secure messaging, and a confidential helpline to individuals who are concerned about their sexual thoughts, feelings, or behavior toward children. Lately, a self-help section has been established offering information concerning sexual interest in children, facts and consequences of sexual offending, and hints on how to abstain from abusive behaviors. To date, evaluation studies of both web-based programs have not been published. Consequently, programs that specifically aim to prevent child sexual offenses and the consumption of CSAM are scarce. The few existing programs are rather short and do not offer self-assessments regarding sexual fantasies and sexual behavior (including child sexual offense and the consumption of CSAM). However, completion of the assessments can already be regarded as a critical reflection of one’s own sexual interest and sexual behavior [37]. Moreover, especially the feedback after the completion offers clarification about the problematic sexual behavior. In line with that, behavioral changes have been associated with the individual’s perception of their own behavior as “problematic” [38]. The existing programs are mainly available in English, and non–English-speaking individuals have not been addressed so far (except for Gemeinsam statt allein). This is where the innovative potential of Troubled Desire comes in. Troubled Desire was developed at the Berlin Institute of Sexology and Sexual Medicine to provide web-based (1) self-assessments and (2) self-management training modules for individuals with sexual interest in prepubescent and early pubescent children. To reach out to individuals from various countries and regions, Troubled Desire is available in different languages. Troubled Desire aims to prevent child sexual offense and the use of CSAM and to alleviate the distress experienced by those with sexual interest in children.

### Study Aim

The aim of this study was to describe the population who completed the self-assessment within the first 30 months after the launch of the Troubled Desire app. We report on the following characteristics of the users of this app: (1) sociodemographics, including gender, age, and country of origin, and (2) sexual interest and offense characteristics.

## Methods

### Description of the App

Troubled Desire is a web-based app that requires an internet-enabled device and a common web browser. This app is composed of (1) a self-assessment and (2) self-management training modules for individuals with sexual interest in prepubescent and early pubescent children. During this study, 7 different language versions were gradually implemented: English (since October 25, 2017), German (since April 25, 2018), Spanish (since October 1, 2018), Portuguese (since July 12, 2019), French (since July 19, 2019), Hindi (since September 12, 2019), and Marathi (since December 1, 2018). This app was approved by the institutional review board of the Charité-Universitätsmedizin Berlin.

### Self-assessment

**Figure 1:**
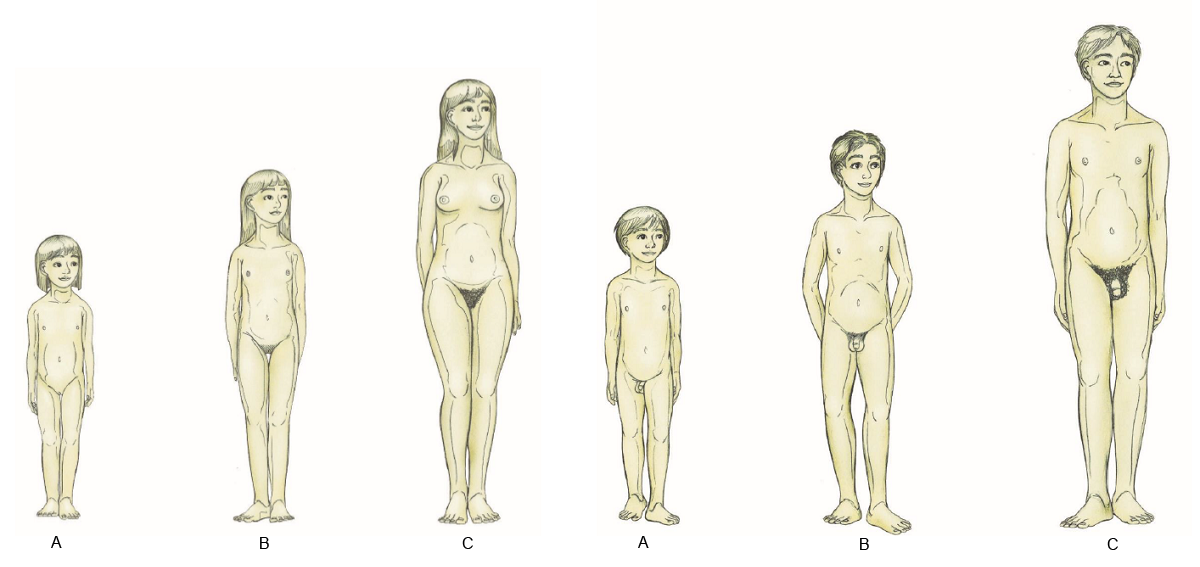
Developmental age categories according to the Tanner stages. A. Tanner stage 1, prepubescent body scheme; B. Tanner stages 2 and 3, early pubescent body scheme; C. Tanner stages 4 and 5, adult body scheme.

At the outset of the self-assessment, the user is provided with a unique personal and randomly generated 9-letter long session ID that allows them to interrupt and continue the session and correct or update the given answers at any time. The self-assessment contains 100-280 questions, which depends on the given answers as there are subquestions depending on the answers given for the main questions, thereby leading to an extended assessment. The processing time takes about 15-30 minutes. The assessment contains questions concerning the sociodemographic background, sexual interest regarding the age and gender of the fantasized partner, the use of CSAM, child sexual offense, and judicial status (if applicable). Illustrations of the Tanner stages (illustrations of the physical development from prepubescent children to adults [39,40]; Figure 1) are displayed throughout the assessment to facilitate responses relating to the age of the sexually fantasized partner. The assessment is solely based on self-report. After completion of the self-assessment, the user receives feedback concerning his/her sexual interest (ie, pedophilic and hebephiliac sexual interest) and sexual behavior, including the child sexual offense and the use of CSAM, if reported.

### Self-management Training Modules

The self-management training modules are based on the Berlin Dissexuality Therapy Program [12] and address major risk factors for child sexual offense and the use of CSAM. The training modules are psychoeducational and give instructions on, for example, how to exercise emotion regulation, train awareness and mindfulness, or how to integrate the sexual interest in children in the self-concept.

### Data Collection

Data from the self-assessment are saved anonymously on a server at the Charité-Universitätsmedizin Berlin. No internet protocol addresses or server log files are stored. This web-based app has not run web analytics processes. Therefore, there was no count of the actual visitors of the website. The user’s country of origin, date, and time of access were automatically identified upon entry by an internally hosted module. Countries with 100,000 inhabitants or less were identified as “anonymous” by the module. This was done to increase granularity so that the identification of individual users was not possible, since the identification of individuals would have been possible with a unique combination of characteristics such as number of children together with the precise profession and knowledge about the country of origin.

### Dissemination of the App

A strategy for web-based and offline advertising was implemented with a focus on Germany and India. The focus was due to 2 reasons: (1) the availability of a treatment infrastructure that users of Troubled Desire might turn to in case of increased need for assistance and (2) an already existing recruitment strategy with high reach from which Troubled Desire could benefit by being embedded. Web-based activities included advertising on partner websites and social media as well as warnings in search engines and peer-to-peer networks. Offline activities included advertising on television, public transport, and billboards. Media coverage also played a role in increasing awareness of the service. The app was promoted by the prevention network “Kein Täter werden” (or Don’t offend [10]) in Germany and “Program for Primary Prevention of Sexual Violence” in India [41], which offer specialized outpatient treatment for people with sexual interest in children.

### Measures

This study investigated the selected questions from the self-assessment. Besides sociodemographic information, the following key variables were examined: (1) presence and absence of sexual interest in children (ie, pedophilic and hebephiliac sexual interest), (2) distress, or (3) trouble due to the given sexual interest, (4) child sexual offense, (5) CSAM consumption, and (6) judiciary status. Sexual interest in children was assessed with dichotomous (yes/no) questions regarding the presence of sexually arousing fantasies of prepubescent children (attracted by the body scheme of Tanner stage 1, ie, pedophilia) or early pubescent children (attracted by the body schemes of Tanner stages 2 and 3, ie, hebephilia) for at least 6 months. This time specification refers to the time criterion for pedophilia of the fifth edition of the Diagnostic and Statistical Manual of Mental Disorders [4]. The following sample item was asked to assess the presence of sexual interest in children: “During masturbation, I find childlike girls/girls with a prepubescent body type (ie, no pubic hair or developed breasts) sexually arousing for at least six months.” Sexual interest in children was counted as yes if users either reported sexual interest in prepubescent children or early pubescent children or both (regardless of gender). Sexual interest in children was counted as no if users negated sexual interest in both prepubescent children and early pubescent children (ie, exclusive sexual interest in adults, attracted to the body schemes of Tanner stages 4 and 5). Distress and trouble due to the stated sexual interest was assessed with 2 dichotomous (yes/no) questions (Do these fantasies cause you any distress? or Do these sexual desires cause you any trouble in your romantic, social, or professional life?). To assess child sexual offense, 10 questions were analyzed with increasing gravity of the abuse behavior. Questions ranged from taking nude pictures of a child (I have engaged sexually with a child, for instance, taking nude photos/videos, watching pornography together, talking about sex, etc) and making a child perform sexual acts (I have made a child perform sexual acts either on himself/herself or with another child while I was watching) to penetrating a child (I have put my penis into a child’s butt). To answer the questions, users had to select 1 out of the 4 provided options: (1) never, (2) some time ago, (3) recently, and (4) currently. For this study, if any child sexual offense question was answered with (2) some time ago, (3) recently, or (4) currently, the child sexual offense was counted as yes. If every child sexual offense question was answered with (1) never, the child sexual offense was counted as no. Furthermore, in the presence of the reported child sexual offense, lifetime child sexual offense ([2] some time ago, [3] recently, [4] currently) was differentiated from recent child sexual offense ([4] currently). We analyzed 4 dichotomous (yes/no) questions to assess lifetime consumption of CSAM. Questions ranged from children in erotic poses (Have you used depictions of children in clothed, lightly clothed, or naked in erotic/arousing/provocative poses?) to depictions of sexual acts (Have you used depictions of sexual acts with children and adult/adults?). Four similar questions were asked to assess the consumption of CSAM within the last 6 months (eg, Have you used depictions of children in clothed, lightly clothed or naked in erotic/arousing/provocative poses within the last six months?). If any lifetime CSAM question was affirmed, lifetime CSAM consumption was counted as yes. If any question on CSAM consumption within the last six months was affirmed, recent CSAM consumption was counted as yes. If child sexual offense (lifetime or recent) or CSAM consumption (lifetime or recent) was reported, a question followed enquiring users to indicate whether the offense or the offenses had been detected by legal authorities (Have you ever been in contact with the legal authorities for owning/using/distributing such material? or Have you ever been in contact with the legal authorities for child sexual abuse?). Three options were provided to answer the questions: (1) no, (2) prosecuted, and (3) sentenced. Furthermore, to assess the user’s country of origin, we analyzed data, which were automatically identified by an internally hosted module.

### Users

Users refer to individuals who answered at least one question of the self-assessment and therefore left at least one data point. We focused our analyses on these users who completed the assessment within the first 30 months after the launch (October 25, 2017 to April 25, 2020). We included users irrespective of whether they engaged in the self-management modules or not. Users were able to access the self-assessment from anywhere (not just in the countries based on the currently available 7 languages) and choose their preferable language option. Users of all predetermined age categories (see Statistical Analysis) were included in this study. Accordingly, the following inclusion criteria were applied: completion of the self-assessment within the first 30 months after the launch and fully completed assessment.

### Statistical Analysis

Data were analyzed using SPSS Statistics Version 25.0 (IBM Corp). Data of the user characteristics were analyzed descriptively. Pearson chi-square tests were employed to assess group differences (users with sexual interest in children vs users without sexual interest in children). The statistical level of significance was set at .05. As participants indicated their age by predetermined age categories ranging from “14-15 years” to “70 years and above,” age results were reported by the mode and the respective full range.

## Results

A total of 7496 users started the self-assessment of Troubled Desire within the first 30 months after the launch. The assessment was fully completed by 4161 users. Of these, 90.9% (3783/4161) were males, 6.9% (289/4161) were females, and 2.1% (89/4161) did not assign to the binary gender system (referred to as nonbinary). Approximately 80.0% (3329/4161) of the users were younger than 40 years. The most frequently chosen age category was “19-21 years” (mode). The most common geographical accesses were from Germany (2277/4161, 54.7%) followed by the United States (474/4161, 11.4%), France (130/4161, 3.1%), and the United Kingdom (131/4161, 3.1%, Table S1 of Multimedia Appendix 1).

Approximately 78.9% (3281/4161) of the participants reported sexual interest in children (ie, pedophilic and hebephiliac sexual interest; Table 1). Users with sexual interest in children were significantly more likely to report distress (*Χ*^2^_1_ [N=4161]=758.1, *P*<.001) and trouble (*Χ*^2^_1_ [N=4161]=305.2, *P*<.001) than users who did not have sexual interest in children. We observed a significant relationship between sexual interest and sexual offense behavior. Users with sexual interest in children were more likely to report both lifetime (*Χ*^2^_1_ [N=4161]=631.4, *P*<.001) and recent CSAM consumption (*Χ*^2^_1_ [N=4161]=578.1, *P*<.001) than users without sexual interest in children. Additionally, both lifetime (*Χ*^2^_1_ [N=4161]=151.9, *P*<.001) and recent child sexual offense (*Χ*^2^_1_ [N=4161]=13.1, *P*<.001) were significantly more common among users with sexual interest in children than among users without sexual interest in children.

Sexual offenses have mainly been undetected by legal authorities. Comparing both groups, the chi-square test showed that users with sexual interest in children were less likely to be in contact with the justice system because of child sexual offense (*Χ*^2^_1_ [n=1530]=5.2, *P=*.02) than were users without sexual interest in children. The proportion of users who had contact with the legal authorities did not differ by sexual interest (*Χ*^2^_1_ [n=2626]=0.5, *P=*.83).

**Table 1:** Sexual interest and offense characteristics of the users of the Troubled Desire app (N=4161).

Characteristics		Users with sexual interest in children^a^ (n=3281), n (%)	Users without sexual interest in children (n=880), n (%)	Chi-square *(df)*	*P* value
**Sexual interest**					
	Distress due to sexual interest	2405 (73.3)	200 (22.7)	758.1 (1)	<.001
	Trouble due to sexual interest	1673 (51.0)	159 (18.1)	305.2 (1)	<.001
**Offense characteristics**					
	Consumption of child sexual abuse material, lifetime	2390 (72.8)	236 (26.8)	631.4 (1)	<.001
	Consumption of child sexual abuse material, recent	2157 (65.7)	180 (20.5)	578.1 (1)	<.001
	Prosecuted or sentenced	388 (16.2)	37 (15.25)	0.5 (1)	.83
	Child sexual offenses, lifetime	1363 (41.5)	167 (19.0)	151.9 (1)	<.001
	Child sexual offenses, recent	163 (5.0)	19 (2.2)	13.1 (1)	<.001
	Prosecuted or sentenced	128 (9.4)	25 (15.0)	5.2 (1)	.01

^a^Pedophilic and hebephiliac sexual interest.

## Discussion

### Overview of the Findings

This study presents the first assessment of Troubled Desire, a web-based program that offers anonymous and confidential (1) self-assessments and (2) self-management training modules for individuals with sexual interest in children (ie, pedophilic and hebephiliac sexual interest). The data of the users within the first 30 months after the launch of Troubled Desire were analyzed. We compared users who reported sexual interest in children with users who did not report sexual interest in children (ie, exclusive sexual interest in adults). The following main findings were derived: (1) distress and trouble due to sexual interest and (2) child sexual offenses and the use of CSAM were significantly more common among individuals with sexual interest in children than among individuals without sexual interest in children (*P*<.001), and (3) the vast majority of child sexual offenses and CSAM offenses were not known to legal authorities.

Pedophilia has been associated with clinically relevant distress [42], anxiety [43-45], stigmatization, and discrimination [15,46]. This is in accordance with our results that point to a significant association between sexual interest in children and the reported distress and trouble in the romantic, social, or professional life. Jahnke [47] has shown that individuals with sexual interest in children and stigma-related distress rarely seek therapeutic help, which increases the likelihood of offending or reoffending. For those individuals, a web app might provide a good opportunity to receive low-threshold support. However, considering that guided treatment is superior to unguided treatment [24], we cannot yet state whether such a web-based app is sufficient for reducing the risk of offending or reoffending and for increasing the mental well-being. Future studies will be necessary to examine the potential and weaknesses of guided treatments, particularly regarding the reduction in the offending behavior. Child sexual offenses and the use of CSAM were significantly more common among individuals with sexual interest in children. This is in line with the notion that pedophilia and hebephilia can be regarded as the major risk factors for committing child sexual offense and for the consumption of CSAM [6,7].

First results of the treatment program of the Berlin-based outpatient treatment service for individuals with sexual interest in children revealed a significant reduction in the risk factors associated with the offending behavior (eg, offense-supportive cognition, emotional deficits) and a drop in the offending behavior on a descriptive level [10]. These initial results are promising, and considering user accesses from more than 80 countries, an international expansion is suggested. The need for expansion is also underlined by the fact that access rates highly differed. As the majority of the accesses stem from Germany (2277/4161, 54.7%), it seems to be easier to reach out to the target group when an established project structure with a history of media campaigning and public awareness already exists (eg, the prevention network “Kein Täter werden”[10]). In India, this app has indeed been promoted by the “Program for Primary Prevention of Sexual Violence” [41]. However, this treatment service has just been developed recently. Additionally, we do not yet know whether transferability of the treatment approach to other countries with different cultural backgrounds is feasible. Therefore, it might be interesting to investigate which resources might be necessary and which barriers must be eliminated to apply the treatment approach to other countries.

Most of the sexual offenses remained undetected. This finding emphasizes that disclosed cases of child sexual offending represent only a small portion of the total offenses. The actual number of the cases are much higher. The high number of undetected cases is alarming and stresses that it is indispensable to invest in global preventive measures. Referring to our results, it is questionable why users with sexual interest in children were significantly less likely to be in contact with the justice system because of child sexual offense than users without sexual interest in children. It might be speculated that those users were *better* at covering up their offenses or might have intimidated their victims to a great degree. Additionally, it might be hypothesized that their offenses were acted according to an intended procedure, whereas users without sexual interest in children might have abused rather impulsively.

In this study, almost half of the users (3335/7496) accessed and started the self-assessment without completing it. It remains unclear why these users were intrigued in the beginning but dropped out at some point. Conceivable reasons are that some participants aborted the assessment because they felt deterred by explicit sexual questions or were afraid of being detected. Future evaluations of Troubled Desire should therefore focus on the dropouts and whether certain questions might have led to more dropouts. More male users than female users (and nonbinary users) accessed Troubled Desire. This indeed coincides with the findings in existing literature showing that sexual interest in children is more common in men than in women [48,49]. At the same time, this also underlines the fact that sexual interest in children is also found in women. Future evaluations should focus more on possible gender differences and on the subgroup of nonbinary users in terms of sexual interest or sexual behavior.

### Limitations of This Study

This study has a few limitations. First, Troubled Desire is anonymous and does not store internet protocol addresses or server log files. Therefore, we cannot verify whether the given data in the self-assessment is related to a real-life person. Second, we were not able to verify whether users truthfully answered the questions. It might have been possible that users suppressed or whitewashed certain information. However, as the self-assessment was anonymous, we do not expect a major confoundment here. Third, we were not able to operationalize recent child sexual offenses and recent CSAM consumption similarly, as questions within the self-assessment varied for both question blocks. The questions on child sexual offenses differentiated between (1) never, (2) some time ago, (3) recently, and (4) currently. Recent child sexual offense was accounted as yes, if any child sexual offense question was answered with (4) currently. However, recent CSAM was assessed with a specified time frame, that is, within the last 6 months. We can, therefore, not certainly state that answers to those questions refer to the same time period.

### Conclusions

Troubled Desire was able to reach out to individuals with sexual interest in children. User characteristics confirmed findings in prior studies with evidence for more distress and child sexual offending behavior (ie, child sexual offenses, consumption of CSAM) in individuals with sexual interest in children. Though its prospective relevance in the prevention of child sexual offending remains to be seen, we think that Troubled Desire has, nonetheless, the potential for providing self-assessments and self-assessment training modules for those who do not seek help or cannot obtain help from health care professionals for reasons of mandatory reporting, fear, or lack of accessibility.

## Appendix

Multimedia Appendix 1 Country of origin of the users.

## Abbreviations

CSAM: child sexual abuse material
