# Supplementary material for: Characteristics of the Users of Troubled Desire, a Web-Based Self-management App for Individuals With Sexual Interest in Children: Descriptive Analysis of Self-assessment Data
Source: JMIR Ment Health. 2021 Feb 19;8(2):e22277. doi: 10.2196/22277 (PMC7935650; doi:10.2196/22277)
Supplement: Multimedia Appendix 1 [file mental_v8i2e22277_app1.docx]

**Supplementary table**

**Table S1.** Users country of origin (N=4161).

| Country of origin | | Users | Country of origin | | Users |
| --- | --- | --- | --- | --- | --- |
| **Africa, n (%)** | |  | **Europe, n (%)** | |  |
|  | Egypt | 5 (0.1) |  | Denmark | 13 (0.3) |
|  | Liberia | 7 (0.2) |  | Finland | 8 (0.2) |
|  | Mauritius | 1 (0.0) |  | France | 130 (3.1) |
|  | Morocco | 2 (0.0) |  | Georgia | 1 (0.0) |
|  | Namibia | 2 (0.0) |  | Germany | 2277 (54.7) |
|  | South Africa | 10 (0.2) |  | Greece | 8 (0.2) |
|  | Zambia | 1 (0.0) |  | Hungary | 2 (0.0) |
|  | Total | 31 (0.6) |  | Ireland | 18 (0.4) |
| **Asia, n (%)** | |  |  | Italy | 19 (0.5) |
|  | Bahrain | 2 (0.0) |  | Latvia | 1 (0.0) |
|  | Cambodia | 2 (0.0) |  | Lithuania | 1 (0.0) |
|  | China | 4 (0.1) |  | Luxembourg | 6 (0.1) |
|  | Hong Kong | 2 (0.0) |  | Moldova | 8 (0.2) |
|  | India | 91 (2.2) |  | Netherlands | 62 (1.5) |
|  | Indonesia | 13 (0.3) |  | Norway | 5 (0.1) |
|  | Iran | 2 (0.0) |  | Poland | 6 (0.1) |
|  | Israel | 7 (0.2) |  | Portugal | 21 (0.5) |
|  | Japan | 5 (0.1) |  | Romania | 16 (0.4) |
|  | Korea, Republic of | 3 (0.1) |  | Russia | 14 (0.3) |
|  | Malaysia | 4 (0.1) |  | Serbia | 1 (0.0) |
|  | Maldives | 1 (0.0) |  | Slovakia | 5 (0.1) |
|  | Pakistan | 2 (0.0) |  | Slovenia | 11 (0.3) |
|  | Philippines | 2 (0.0) |  | Spain | 25 (0.6) |
|  | Qatar | 1 (0.0) |  | Sweden | 18 (0.4) |
|  | Singapore | 6 (0.1) |  | Switzerland | 75 (1.8) |
|  | Sri Lanka | 2 (0.0) |  | Ukraine | 13 (0.3) |
|  | Taiwan | 3 (0.1) |  | United Kingdom | 131 (3.1) |
|  | Thailand | 8 (0.2) |  | Total | 3013 (73.3) |
|  | Turkey | 6 (0.1) | **North America, n (%)** | |  |
|  | United Arab Emirates | 1 (0.0) |  | Canada | 88 (2.1) |
|  | Vietnam | 3 (0.1) |  | Costa Rica | 1 (0.0) |
|  | Yemen | 1 (0.0) |  | El Salvador | 1 (0.0) |
|  | Total | 171 (3.7) |  | Honduras | 1 (0.0) |
| **Australia, n (%)** | |  |  | Nicaragua | 1 (0.0) |
|  | Australia | 64 (1.5) |  | United States | 474 (11.4) |
|  | New Zealand | 6 (0.1) |  | Total | 568 (13.5) |
|  | Papua New Guinea | 1 (0.0) | **South America, n (%)** | |  |
|  | Total | 71 (1.6) |  | Argentina | 13 (0.3) |
| **Europe, n (%)** | |  |  | Brazil | 39 (0.9) |
|  | Albania | 1 (0.0) |  | Chile | 23 (0.6) |
|  | Algeria | 1 (0.0) |  | Colombia | 3 (0.1) |
|  | Austria | 81 (1.) |  | Mexico | 13 (0.3) |
|  | Belarus | 3 (0.1) |  | Paraguay | 1 (0.0) |
|  | Belgium | 16 (0.4) |  | Peru | 1 (0.0) |
|  | Bulgaria | 4 (0.1) |  | Uruguay | 2 (0.0) |
|  | Croatia | 2 (0.0) |  | Total | 95 (2.2) |
|  | Czech Republic | 8 (0.2) | **Anonymous, n (%)** | | 212 (5.1) |

Notes: Anonymous refers either to countries with less than 100.000 inhabitants or when automatic identification was not possible.

Abbreviations: n=subsample size
